# Fiber Pathways of Attention Subnetworks Revealed with Tract-Based Spatial Statistics (TBSS) and Probabilistic Tractography

**DOI:** 10.1371/journal.pone.0078831

**Published:** 2013-11-04

**Authors:** Haitao Ge, Xuntao Yin, Junhai Xu, Yuchun Tang, Yan Han, Wenjian Xu, Zengchang Pang, Haiwei Meng, Shuwei Liu

**Affiliations:** 1 Research Center for Sectional and Imaging Anatomy, Shandong University School of Medicine, Jinan, Shandong, China; 2 Department of Radiology, Affiliated Hospital of Medical College, Qingdao University, Qingdao, Shandong, China; 3 Department of Epidemiology, Qingdao Municipal Center for Disease Control and Prevention, Qingdao, Shandong, China; University of California, San Francisco, United States of America

## Abstract

It has been widely accepted that attention can be divided into three subnetworks - alerting, orienting and executive control (EC), and the subnetworks of attention are linked to distinct brain regions. However, the association between specific white matter fibers and the subnetworks of attention is not clear enough. Using diffusion tensor imaging (DTI), the white matter connectivity related to the performance of attention was assessed by attention network test (ANT) in 85 healthy adolescents. Tract-based spatial statistics (TBSS) and probabilistic diffusion tractography analysis demonstrated that cerebellothalamic tract was involved in alerting, while orienting depended upon the superior longitudinal fasciculus (SLF). In addition, EC was under the control of anterior corona radiata (ACR). Our findings suggest that different fiber pathways are involved in the three distinct subnetworks of attention. The current study will yield more precise information about the structural substrates of attention function and may aid the efforts to understand the neurophysiology of several attention disorders.

## Introduction

Attention refers to the cognitive process of concentrating on the relevant information while ignoring the irrelevant ones. Consistent with Posner's framework of attentional systems, recent brain imaging studies have consistently supported the idea that there are three key distinct subsystems of attention, namely alerting, orienting, and executive control (EC) [1]–[3].Briefly, alerting is defined as achieving and maintaining a state of high sensitivity; orienting is the selection of sensory information; and EC is involved with the process of resolving cognitively incongruent stimuli [4]. Numerous brain imaging studies have indicated that distinct cortical and subcortical areas are engaged in the three subnetworks of attention [5]–[8]. However, it remains largely unknown whether the fiber pathways between these regions are associated with attention function.

Diffusion tensor imaging (DTI) is a powerful tool for the investigation of fiber pathways in vivo. An increasing number of studies have suggested that individual variations in white matter microstructure are specifically associated with individual differences in cognitive functions, such as intelligence [9], [10] as well as working memory [11], [12]. With the extensive use of DTI, many white matter regions have also been reported to be involved in attention function (Table 1).

**Table 1:** A summary of the previous findings of white matter regions associated with attention.

Studies	Samples	Methods	Regions
Nestor et al. (2007)	18	ROI	Cingulum
Niogi et al. (2008)	66	ROI	ACR
Kubicki et al. (2009)	36	ROI	Cingulum
Konrad et al. (2010)	71	VBM	SLF
Mamah et al. (2010)	72	ROI	ATR
Niogi et al. (2010)	26	ROI	PLIC, splenium of CC, ACR
Takahashi et al. (2010)	38	VBM	Cingulum, cerebellar peduncle
Tang et al. (2010)	45	VBM	ACR
Umarova et al. (2010)	26	VBA	SLF, AF, IFOF
Thiebaut de Schotten et al. (2011)	20	ROI	SLF
Urbanski et al. (2011)	24	ROI+VBM	ALIC
Chechlacz et al. (2012)	59	ROI	SLF,IFOF,ILF
Tartaglia et al. (2012)	27	ROI	Cingulum
Yin et al. (2012)	59	TBSS	ACR
Wu et al. (2012)	50	ROI	Frontostriatal tract
Klarborg et al. (2013)	76	ROI	SLF
Asami et al. (2013)	51	ROI	MLF
Vallar et al. (2013)	7	ROI	SLF

ACR: anterior corona radiata, AF: arcuate fasciculus,ALIC: anterior limb of internal capsule, ATR: anterior thalamic radiation, CC: corpus callosum, IFOF: inferior fronto-occipital fasciculus, MLF: middle longitudinal fasciculus, PLIC: posterior limb of internal capsule, SLF: superior longitudinal fasciculus.

The structure–function correlation between alerting and the posterior limb of the internal capsule has been reported [13]. The splenium of the corpus callosum [13] and left cingulum [14] have been found to be linked with orienting function. EC has been reported to be mediated by anterior corona radiata (ACR) and anterior thalamic radiation [13], [15], [16]. Visuospatial attention can also be divided into the dorsal and ventral frontoparietal networks [17]. The dorsal network was reported to link temporoparietal cortex with superior longitudinal fasciculus (SLF) and the arcuate fascicle (AF), while the structural connection for the ventral network was inferior fronto-occipital fascicle (IFOF) [18]. There was also a work confirmed that right SLF was involved in visuospatial attention [19]. Sustained attention has also been reported to be associated with the right cingulum, bilateral cerebellar peduncles [20], right SLF and superior parietal white matter microstructure [21]. However, within the results, there were confusions about which fibers were involved in attention. Also, most of the microstructure of white matter fibers within a set of regions of interest (ROIs) was measured by outlining the ROIs or by placing a fixed geometric shaped ROI manually. As ROI is based on the priori knowledge, the reliability and repeatability cannot be guaranteed.

Tract-based spatial statistics (TBSS) is a new developed method which allows voxelwise statistical comparison between individual subject's DTI data for whole brain analysis [22]. It was developed to alleviate registration and spatial smooth problems related to conventional voxelwise analysis. TBSS compares individual subject's fractional anisotropy (FA) values within the skeletons of white matter instead of the entire white matter. On the other hand, fiber tractography using non-invasive brain imaging data can trace the anatomical connections between different brain regions in vivo. The combination of TBSS and fiber tractography will provide a better understanding about the anatomical substrates of the attention function.

In our previous work, we have found that FA in frontal ACR played a crucial role in EC function [23]. Due to the smaller sample size, we failed to reveal the structural underpinnings for alerting and orienting. Based on previous literatures, we argued that the three subnetworks of attention depended on different white matter tracts. In the present study, we enlarged the sample size and tried to investigate the white matter fibers involved in the three subnetworks of attention by TBSS and probabilistic tractography analyses. Our study may be valuable towards the understanding of the neural substrates of attention and the neurophysiology of several attention disorders.

## Materials and Methods

### Subjects

Forty-three healthy adolescent subjects (23 males, 20 females, mean age: 17.17±2.6 years) were recruited in this study. In order to avoid any potential “double dipping” in the analysis of later data processing, another forty-two age- and gender-matched subjects (23 males, 19 females, mean age: 17.26±2.5 years) were also recruited [24]. All subjects in the two groups were Chinese speakers and had no neurological or psychiatric history. They were right-handed measured with Edinburgh Handedness Inventory [25]. The accuracy of ANT performance for each subject was not less than 80% and the scores of the three subnetworks were positive. Written informed consent was obtained from their parents on the behalf of each subject. This study was approved by the Ethic Committee of Shandong University.

### ANT task

**Figure 1:**
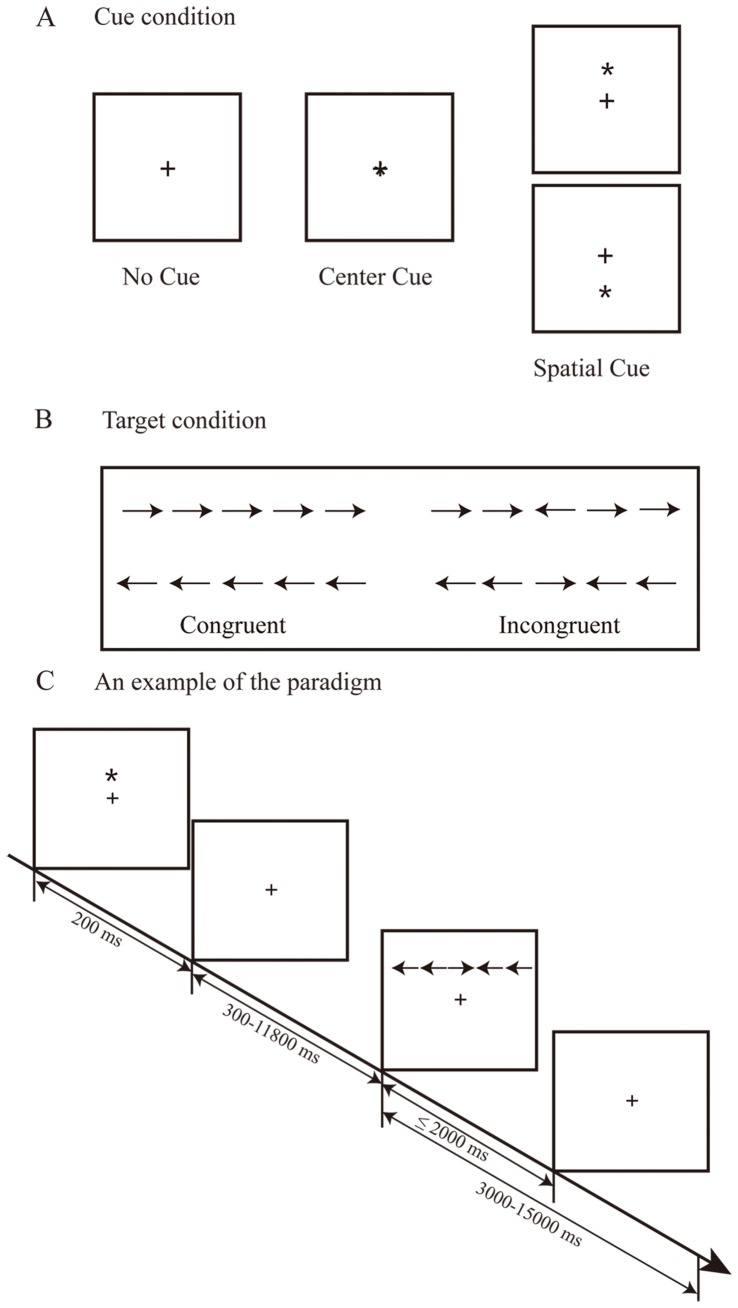
The attention network task (ANT) paradigm. (A)The three cue conditions in ANT. (B)The two target conditions in ANT. And (C) an example of the ANT paradigm: a spatial cue is presented followed by an incongruent target condition.

Attention network test (ANT) was used as the cognitive task in this study to assess the response time (RT) for the three subnetworks of attention [26]. The test was comprised of three cue conditions (no cue, center cue, spatial cue) and two target conditions (congruent, incongruent). Each subject performed a total of six blocks of trials, each block consisting of 36 trials plus 2 buffer trials at the beginning and lasting 5 min 42 s. In each block, the six trial types (3 cue conditions by 2 target conditions) were presented in a predetermined counterbalanced order (see Figure 1). All trials presented a target stimulus, either above or below the fixation cross. All subjects were instructed to press a button as quickly and accurately as possible to make a left-right determination. They were trained before the formal task. Stimulus presentation and behavioral response collection was performed using E-Prime (Psychology Software Tools, Pittsburgh, PA).

### MRI acquisition

MRI scans were performed on a 3.0 Tesla GE Signa scanner (General Electric Medical Systems, Milwaukee, WI). DTI images were acquired with a single shot, spin-echo echo planar imaging sequence (TR/TE = 14000/75.1 ms, acquisition matrix = 96×96, field of view (FOV) = 250×250 mm^2^, slice thickness = 2.6 mm, with no gap). The DTI scans included 30 directions with non-collinear diffusion gradients (b = 1000 s/mm^2^) and 3 non-diffusion-weighted (b = 0 s/mm^2^). Parallel imaging was employed using the Array Spatial Sensitivity Encoding Technique (ASSET) with an acceleration factor of 2. For each subject 56 axial slices were acquired and the DTI scans were repeated two times to increase signal-to-noise ratio (SNR). The time for total scans was about 16 min 20 s.

### Data processing

#### ANT data analysis

The total accuracy of each subject was calculated and the subjects with high error rates (>20%) or negative behavioral scores were excluded in this study. In addition, the trials with incorrect responses or with RTs longer than 1500 ms or shorter than 200 ms were also excluded. We also removed responses following erroneous ones to avoid post-error slowing effect. Since RTs were not normally distributed, we used median RT per condition as raw scores. The accuracy for each of the six trial types was also calculated. Finally, instead of the conventional subtraction measure, we used ratio scores of alerting, orienting, and EC to definite the effects of three attention subnetworks [27]. The formulas were as follows:

Alerting effect = 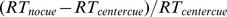


Orienting effect = 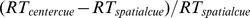


EC effect  = 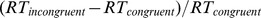


#### DTI data analysis

All DTI data were processed using FSL toolbox (FMRIB Software Library, http://www.fmrib.ox.ac.uk/fsl) [28]. Firstly the diffusion data were corrected for eddy currents and head motion and the two acquisitions were averaged. The averaged images were masked to remove skull and non-brain tissue using the FSL Brain Extraction Tool (BET) [29]. Afterwards, the diffusion tensor model was fitted to create FA maps using FMRIB's Diffusion Toolbox (FDT) [30].

Whole brain voxelwise statistical analysis of FA maps of the both two groups was performed by TBSS separately [22]. Briefly, all subjects' FA maps were nonlinearly aligned into a standard space. Next, the mean FA image was created and thinned to create a mean FA skeleton which represented the cores of all tracts common to the group. The mean FA skeleton was further thresholded by a FA value of 0.2 to exclude the skeleton voxels which may contain gray matter or cross-subject image misalignment. Then each subject’s FA map was projected onto the mean FA skeleton to generate an individual FA skeleton map in MNI152 standard space (1×1×1 mm^3^).

#### Statistical analysis

Voxel-based correlation analyses were conducted to examine the relationships between FA skeleton maps of the first group and attention behavioral scores with gender and age as the covariates of no interest using SPM8. The correction for multiple comparison was performed using Monte Carlo simulation implemented in AFNI (3dClustSim, http://afni.nimh.nih.gov). Clusters larger than 10 voxels (mm^3^) at a threshold of *p*<0.05 (with a peak voxel of *p*<0.001) were considered significant. The ICBM-DTI-81 White-Matter Labels Atlas was used to identify the anatomical location of significant clusters [31].

### Probabilistic tractography

To provide further information with regard to the white matter pathways mediating attention performance, we used the clusters of white matter showing significant correlation with attention behavioral scores as the seed masks to perform probabilistic tractography for the second group in native space [30]. A multi-fiber model was used to fit to diffusion data at each voxel to allow tracking fibers [32]. Fiber tracking was performed from all voxels within each seed mask (5000 streamline samples per seed voxel, 0.5 mm step lengths, curvature threshold = 0.2). Connectivity distributions from the seed masks would be generated and all brain voxels had a value representing the connectivity value between that voxel and the seed mask.

The results of the probabilistic tractography for each subject were binarized and summed before thresholded to 80% to create probabilistic fiber masks. The JHU white matter tractography atlas served as a structural reference [33]. The product of the probabilistic tractography for each subject was a connectivity map showing the number of streamline samples arrived from the seed voxels. The connectivity map from each subject was binarized and summed up to create a probabilistic map in MNI space. The probabilistic map was overlaid on the fiber atlas in FSLView to check whether the two images were similar. In addition, the mean FA value of each probabilistic fiber for each subject was extracted to perform correlation analysis to further explore the relationships between white matter fibers and attention performance.

## Results

### Attention performance

The mean accuracy of ANT performance for all the subjects was 97%. There was no significant difference in the ratio scores of alerting, orienting, EC and accuracy between the two groups (Table 2). There were also no significant gender differences in the ratio scores of alerting, orienting, EC and overall accuracy within each group (*p>*0.05). Correlation analysis showed that only the correlation between alerting and orienting was significant (*p*<0.05).

**Table 2:** Demographics and attentional performance of the two groups.

	Group 1	Group 2	*p* value
Demography			
Age (years)	17.17±2.63	17.26±2.45	0.81
Gender (M/F)	23/20	23/19	0.91^a^
Attention			
Alerting	0.060±0.001	0.055±0.001	0.37
Orienting	0.095±0.002	0.099±0.003	0.75
EC	0.167±0.004	0.165±0.002	0.88
Accurate	0.964±0.001	0.971±0.001	0.34

a
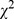
test

### Relationship between white matter anisotropy and attention

#### Alerting

**Figure 2:**
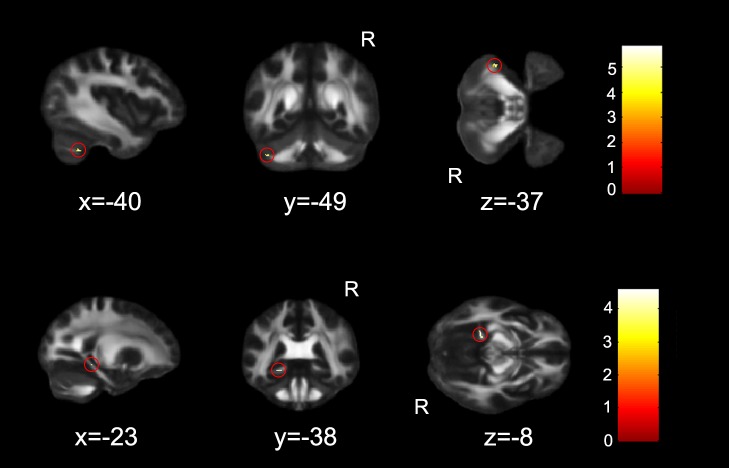
FA skeleton regions where alerting ratio scores showed a significant positive correlation with FA maps (p<0.05, corrected). The region in red circle was identified as the left cerebellar Crus I and cingulum provided by FSL. The coordinates are in MNI standard space. R, right hemisphere.

Multiple regression analysis demonstrated the significant correlation between alerting scores and FA values in the left cerebellar Crus I and the left cingulum (Figure 2, Table 3).

**Table 3:** FA skeleton regions for the correlations between attentional performances and FA skeleton maps.

Regions	Peak MNI coordinate			Peak intensity	Voxels
	x	y	z		
Alerting					
Left Cerebellum	−40	−49	−37	5.81	20
Left Cingulum	−23	−38	−8	4.60	15
Orienting					
Right SLF	34	−24	36	4.17	16
Right Precuneus	27	−55	24	4.66	14
Right Inferior Frontal Gyrus	39	34	7	5.84	12
EC					
Right ACR	25	24	−2	−4.61	12

Clusters were defined at *p*<0.001 and size>10 voxel (mm^3^) corrected by Monte-Carlo simulation. Clusters were ordered by the peak intensity. ACR, anterior corona radiata.

#### Orienting

**Figure 3:**
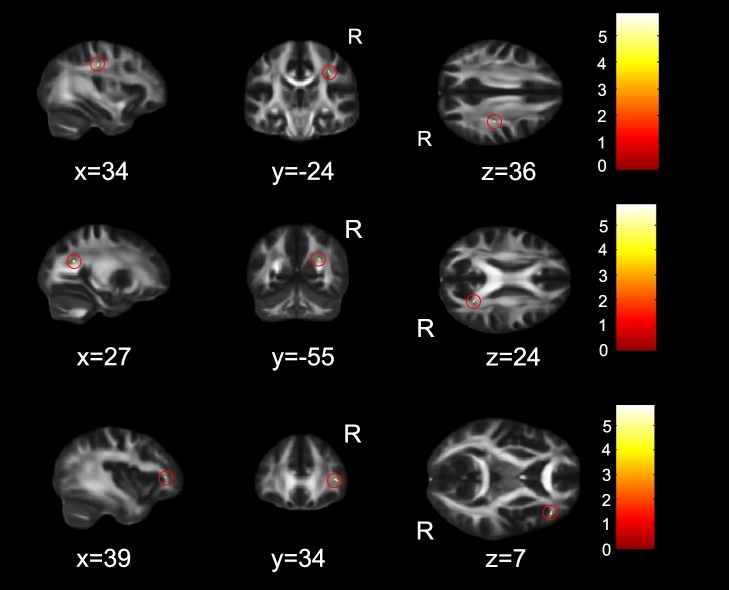
FA skeleton regions where orienting ratio scores showed a significant positive correlation with FA maps (p<0.05, corrected). The regions in red circle were identified as the right superior longitudinal fasciculus (SLF) and white matter beneath the right precuneus and inferior frontal gyrus, respectively. The coordinates are in MNI standard space. R, right hemisphere."

The results of the correlations between the FA values and the orienting scores were reported in Table 3. FA values of the right superior longitudinal fasciculus (SLF) and white matter beneath the right precuneus and inferior frontal gyrus (IFG) correlated significantly with the scores of orienting (Figure 3).

#### EC

**Figure 4:**
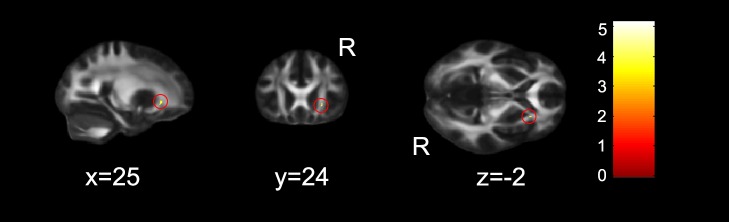
FA skeleton regions where EC ratio scores showed a significant positive correlation with FA maps (*p*<0.05, corrected). The region in red circle was identified as the right anterior corona radiata (ACR). The coordinates are in MNI standard space. R, right hemisphere.

Only one cluster in the right ACR (Table 3, and Figure 4) was survived where FA values correlated with the scores of EC.

### Probabilistic tractography

#### Alerting

**Figure 5:**
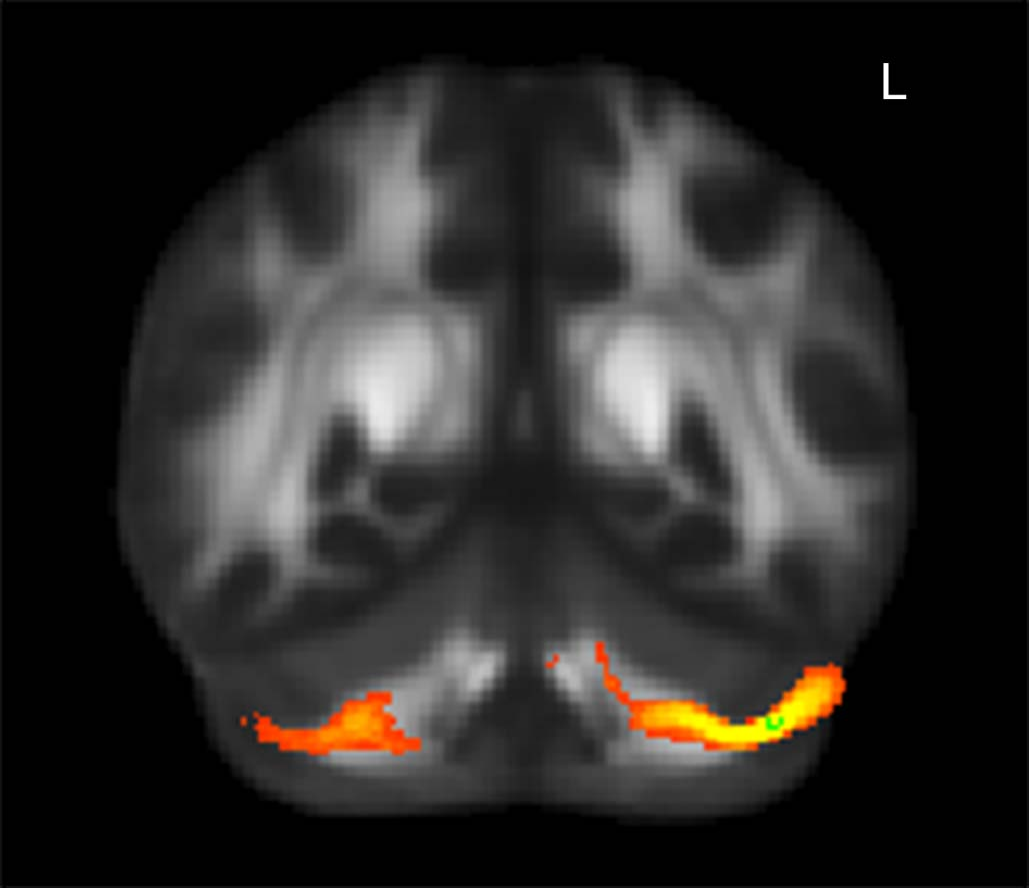
Group probability map generated from the alerting-related region. The red-yellow region was identified as cerebellothalamic tract. The green cluster was the seed mask located in the Crus I.

The probabilistic fiber tracked from the seed mask located in the left cerebellar Crus I was identified to be connected with the thalamus (Figure 5). This fiber arose from cerebellum and entered into thalamus, which was then identified as cerebellothalamic tract. Correlation analysis between mean FA values and the scores of alerting revealed that this fiber was significant correlated with alerting (*p<*0.05) (Table 4). The probabilistic fiber with the left cingulum as the seed mask was identified as the left cingulum. However the FA values of the left cingulum did not showed a significant correlation with the scores of alerting (*p>*0.05).

**Table 4:** Correlations between fiber and attention performance after probabilistic tractography.

Attention subnetworks	Fiber	Voxels	r (*p*)
Alerting	Cerebellothalamic tract	28595	0.230(0.035*)
Orienting	SLF	36787	0.333(0.031*)
EC	IFOF	23946	−0.245(0.113)

IFOF: inferior fronto-occipital fasciculus. * *p*<0.05.

#### Orienting

**Figure 6:**
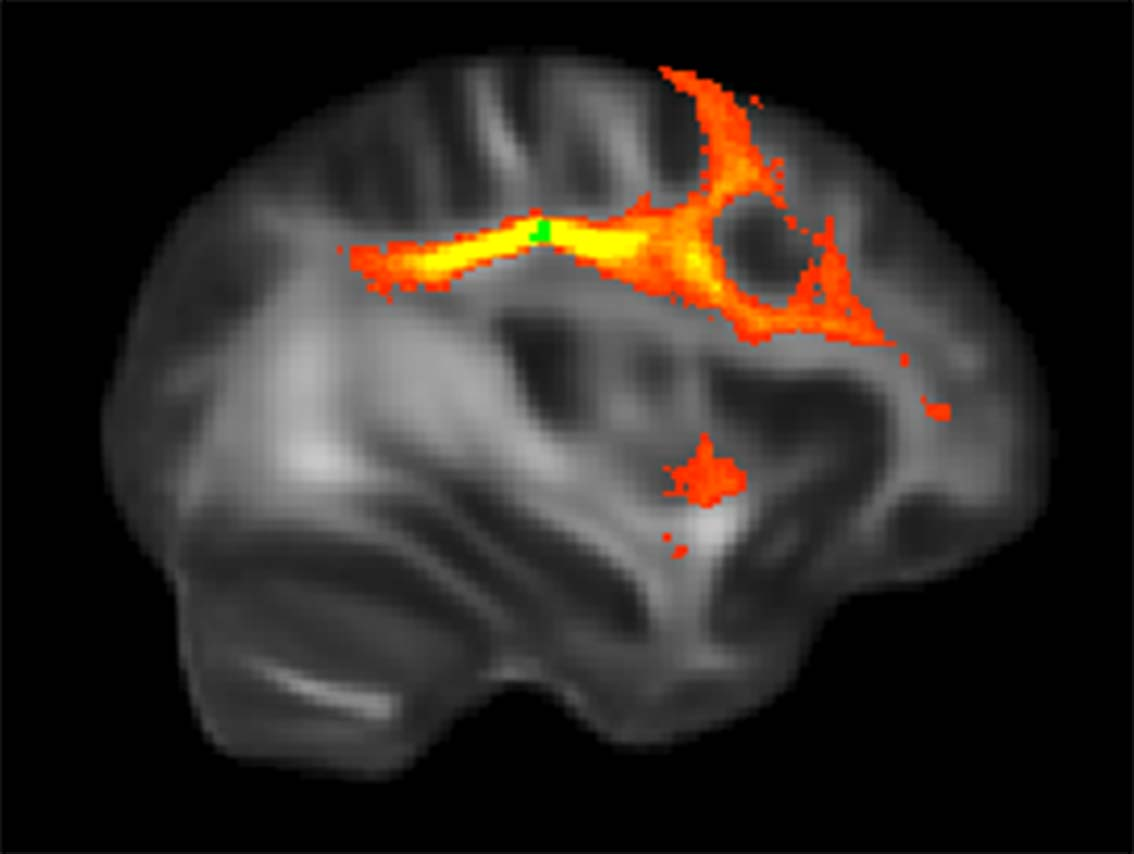
Group probability map generated from the orienting-related regions. The red-yellow region was identified as superior longitudinal fasciculus (SLF). The green cluster was the seed mask located in the right SLF.

Multi-fiber tractography indicated that the connections arising from the right SLF was identified as the right SLF (Figure 6). Correlation analysis showed that the right SLF was highly associated with orienting (*p<*0.05) (Table 4). The fibers tracked from the white matter right precuneus and IFG were identified as the forceps major and right IFOF, respectively. However neither of the correlation analysis between the mean FA values of the two pathways and the scores of orienting was significantly (*p>*0.05).

#### EC

According to JHU white-matter tractography atlas, the pathway tracked from the right ACR was the right IFOF. However, there was no significant correlation between the mean FA values of the tracked fiber bundles and the EC scores (*p>*0.05) (Table 4).

## Discussion

In this study, we used both TBSS and probabilistic tractography approaches to explore the contributions of local white matter microstructure to attention. We proved that specific white matter fibers were associated with the three subnetworks of attention: alerting depended upon the left cerebellothalamic tract, orienting showed a strong involvement in the right SLF, and EC was modulated by the right ACR.

### Attention performance

Our results revealed that alerting was negatively correlated with orienting, indicating an overlap in alerting and orienting subnetworks [34]. It may be due to the incremental effect of spatial as compared with alerting cue. Such correlation was consistent with our previous study [23] and some other studies [35], [36] but was inconsistent with some others [13], [26]. It is speculated that the age and racial differences across studies might account for the controversial results.

### White matter involved in alerting

The cerebellum has traditionally been considered to be mainly involved in motor control. However, in recent years a wealth of evidences have shown that the cerebellum is also critically involved in a range of cognitive functions, such as sensory perception [37], motion perception [38], [39] and emotion [40]. Our findings revealed that alerting was left-lateralized and under the control of the cerebellum through cerebellothalamic tract. The lateralization of alerting was consistent with prior literatures [7], [41]. The cerebellothalamic tract is one of the major efferent fibers of the cerebellum. It connects the deep cerebellar nuclei (dentate nucleus) with the thalamus [42]. We hold the view that the cerebellothalamic tract transmits information from the cerebellum to the thalamus via neural impulses for the sensory systems, and the neural impulses will be transmitted to cerebral cortex to maintain the alerting state via thalamic radiation. Previous studies have confirmed the import role of prefrontal cortex in alerting [6]. The mutual relation between the prefrontal cortex and the cerebello-thalamo-cortical pathway functions also implied that the cerebello-thalamo-cortical pathway was related to the attention function [43]. Lesion studies showed that the pattern of mild attentional dysfunction was consistent with cerebello-thalamo-cortical pathway dysfunction [44]. Animal studies also showed that cerebellothalamic projections may constitute crucial links in different functional channels involved in alerting mechanisms associated with motor behavior [42], [45]. These literatures suggested that cerebellothalamic tract was involved in alerting and the cerebello-thalamo-cortical pathway might be the afferent pathway for alerting function.

### White matter involved in orienting

Superior parietal lobe, temporal parietal junction and the frontal eye fields (FEF) are the key anatomical regions of orienting [6]. In the present study, we showed a correlation between the performance of orienting and the integrity of right SLF. The SLF is the major association white matter pathway that connects the parietal and frontal lobes [46]. The SLF can be divided into three separate components, SLF I, SLF II and SLF III [47]. Among the three components, the SLF II links FEF and temporoparietal cortex and is critical to maintain the state of orienting [19], [48], [49]. Therefore, the SLF was supposed to be the key pathways of orienting networks [18]. Lesion studies also suggested that the disconnection of SLF would lead to the deficits of orientation [50], [51]. These findings suggest that SLF is crucial for the orienting. In addition, our results showed the right hemisphere was responsible for processing the information related to orienting of attentional networks. It is consistent with the classical model of right hemisphere dominance for attention [19], [52].

### White matter involved in EC

Previous studies demonstrated the prefrontal lobe and the anterior cingulate cortex were the EC subnetworks [7], [53], [54]. ACR is an important fiber pathway connecting the frontal lobe to the anterior cingulate cortex [55]. In this study, correlation analysis showed that EC ratio scores were negatively correlated with FA values of the right ACR. Because larger EC score was indicative of worse performance as a result of longer RTs required for conflict resolution [56], the current finding implicated that greater fiber integrity in the right ACR provided positive influence on EC performance. The positive correlation between EC performance and ACR integrity is consistent with previous studies on normal subjects [13], [57]. Further, damage in ACR due to mild [15] or severe [58] trauma could induce deficit in EC of attention. ACR is a complex fiber and consists of a mixture of projection, association, and callosal fibers [55]. In order to identify the fiber pathway involved in EC, we performed probabilistic tractography analysis with the EC-related region as the seed mask. The resulting fiber connected the frontal lobe with the temporal–occipital cortex, and was identified as the IFOF according to the JHU white matter tractography atlas. However, we did not find the significant correlation between the mean FA values of IFOF and the ratio scores of EC, indicating that EC might be underpinned by regional WM or fibers connecting specific brain regions, rather than the long-range, extensively associated fibers. Another possible explanation was that the fibers traced from the ACR region might not be entirely accurate due to the registration bias and crossing-fibers problems.

### Limitations

Although these findings are robust, some limitations of our study also need to be addressed. First, cohorts with narrow age-range were recruited in this study. One pioneer study has found the differences in ANT performance between children and adults, which might be attributed to the progressive myelination of white matter throughout adolescence and early adulthood [59], [60]. However, to date, most studies focus on the neuroanatomical substrates of attention in childhood and adulthood. These findings lack the understanding about how the brain influences attention in adolescents. Because of this, our findings yielded more precise information about the structural substrates of attention in adolescents. Second, although ANT was suitable for obtaining appropriate indexes for the three subnetworks of attention, the alerting and orienting scores were both obtained from the cue conditions, thus their interactions may be incongruent with the separable structural networks of alerting and orienting. In addition, even though fiber tractography is a very informative technique for testing structural connectivity in humans, it is not a direct tool of visualization of the actual anatomy of fibers, only an indirect reconstruction based on measuring the diffusion of water molecules. The tracking results are depended on the acquisition of data (e.g. scan scheme or field strength), biomathematical model (e.g. deterministic versus probabilistic fiber tracking) and the software programs. Besides, fiber tracking is further complicated by the crossing tracts or tract junctions; although we used probabilistic fiber tracking and crossing fiber model to alleviate this problem.

## Conclusion

The present study investigated the fiber pathways involved in the three subnetworks of attention. TBSS and probabilistic tractography analyses demonstrated that distinct fiber pathways are engaged in alerting, orienting and EC of attention. Our study may aid the understanding of the neural substrates of attention.
